# Isokinetic vs. Hand-Held Dynamometry for Assessing Knee Flexor and Extensor Strength in Athletes: Evaluating a Low-Cost Alternative across the Range of Motion

**DOI:** 10.5114/jhk/210346

**Published:** 2025-09-23

**Authors:** Christina Kosti, Athanasios Tsoukos, Iakovos Pelekis, Vassilis Paschalis, Gerasimos Terzis, Gregory C. Bogdanis

**Affiliations:** 1 School of P.E. and Sport Science, National and Kapodistrian, University of Athens, Athens, Greece.; 2 School of P.E. and Sport Science, Democritus University of Thrace, Thrace, Greece.

**Keywords:** angle-specific torque, H/Q ratio, isometric

## Abstract

We explored whether hand-held dynamometry (HHD) could serve as a low-cost, portable alternative for assessing knee flexion and extension strength across the full range of motion. Twelve healthy athletes (7 men, 5 women; aged 21.4–28.5 years) performed maximal concentric (60°/s) and isometric flexion-extension efforts on an isokinetic dynamometer in prone and seated positions. On two separate occasions, peak extension and flexion torque were measured at six knee angles, and values obtained using HHD and isokinetic dynamometry (ISD) were compared at corresponding angles. HHD data demonstrated high reliability at all angles for knee flexion and extension (ICC = 0.812–0.971, p < 0.001). Knee extension torque was similar in HHD and prone ISD isometric measurements at all angles (p > 0.38). Knee flexion torque was similar in HHD and seated ISD isometric measurements at all angles (p ≥ 0.48). The conventional hamstring to quadriceps (H/Q) ratio was similar in HHD and ISD in the seated position for both concentric and isometric measurements (56 ± 11%, 55 ± 8% and 51 ± 10%, respectively, p > 0.792). Angle-specific H/Q ratios from HHD were similar to those obtained by all modes of testing at all knee angles (p > 0.70), except for the knee angle of 10^o^, which was the position where the knee was almost extended. The highest H/Q ratios were observed at the knee angles of 10^o^ and 30^o^ (p < 0.001). HHD is valid and reliable for assessing knee strength, yielding results comparable to ISD across the range of motion of the knee joint. The findings also emphasize the importance of considering angle-specific H/Q ratios.

## Introduction

The evaluation of knee flexion and extension muscle strength is a common practice in sports and exercise settings. It aims to guide the design of personalized strength training programs that address muscular weaknesses and imbalances, reduce the risk of injury, and optimize performance (Chavarro-Nieto et al., 2023; Kellis et al., 2023; Knapik et al., 1991; Suchomel et al., 2016). Assessing knee flexors and extensors is common due to their contribution to performance in most sports, but also because they offer support, maintain stability, and absorb mechanical torque acting on the knee (Bonetti et al., 2017; Daneshjoo et al., 2013). While isokinetic dynamometry (ISD) is considered the gold standard for strength testing (Deones et al., 1994), it is costly, requires expertise to use, and is usually limited to the laboratory or physiotherapy clinic environment (Choi et al., 2023; Green et al., 2018). Hand-held dynamometry (HHD) has been employed as a cheaper and easy-to-use alternative by sport scientists and coaches to accurately evaluate muscle strength at one joint angle, usually the optimal for torque generation (Bohannon, 1990; Green et al., 2018; Macedo et al., 2022).

HHD measurements at a single joint angle have proven reliable for muscle strength measurements across various settings (Bohannon, 1990; Fulcher et al., 2010; Johansson et al., 2015; Martins et al., 2017), with studies showing strong correlations between HHD and ISD measurements in clinical populations (Malouin et al., 1998; Piao et al., 2004), the elderly (Martin et al., 2006; Reed et al., 1993) and more recently in sports populations (Fieseler et al., 2015; Johansson et al., 2015; Juan-Recio et al., 2024; Martins et al., 2017). However, research highlighted limitations such as its reliance on the tester's strength, which could affect its utility in assessing stronger muscle groups like knee flexors and extensors in athletes (Deones et al., 1994; Kelln et al., 2008; Kolber and Cleland, 2005). Another limitation is that almost all studies assessed muscle strength at a single joint angle in a seated position, which may not accurately reflect the peak force or the strength-generating capacity of the knee extensor and flexor muscles throughout the full range of motion (Fulcher et al., 2010; Johansson et al., 2015; Martins et al., 2017).

In addition to peak muscle strength, the hamstrings-to-quadriceps (H/Q) ratio is essential in identifying muscular imbalances that can lead to injury. The H/Q ratio evaluates the balance between knee flexor and extensor strength, during basic joint movements at different angular velocities (Griffin et al., 2000; Hewett et al., 1999; Li and Maffulli, 1996; Orchard, 1997). An imbalance, such as weaker hamstrings relative to the quadriceps, may increase the risk of anterior cruciate ligament (ACL) injuries, as well as hamstring injuries during dynamic activities (Cheung et al., 2012; Croisier et al., 2008; Griffin et al., 2000). The optimal H/Q ratio at an angular velocity of 60°/s has been proposed to range between 60 and 66%, but other values have also been reported (Baumgart et al., 2018; Croisier et al., 2008; Heiser et al., 1984). This ratio has primarily been established based on data from professional athletes in sports such as soccer and American football, but has also been widely adopted across various sports and broader athletic populations, including both male and female individuals. However, traditional H/Q assessments, i.e., dividing the peak flexor by the peak extensor torque are limited in their ability to evaluate strength imbalances across the whole range of motion (ROM).

To address these limitations, the present study measured hamstring and quadricep strength at six different knee angles and two hip joint positions (seated and prone) using both ISD and HHD, to examine strength and imbalances across the entire ROM of the knee joint. Specifically, the study aimed to assess whether HHD, with the aid of a custom-made construct utilizing low-cost tools, could serve as a reliable and accessible alternative to isokinetic dynamometry for measuring knee flexor and extensor strength across various joint positions. Additionally, the study examined strength imbalances between knee flexors and extensors using the H/Q ratio, calculated both in the conventional manner and at each of the measured knee angles. Ultimately, this study aimed to provide sports professionals with a practical and cost-effective method for assessing athletes' muscle strength directly in the training environment, particularly when access to an isokinetic dynamometer is limited, and to support individualized strength training prescriptions aimed at minimizing injury risk and enhancing long-term performance.

## Methods

### Participants

Power analysis (G*Power, version 3.1.9.2; Kiel University, Kiel, Germany) indicated that a minimum sample size of nine participants was needed to detect a medium effect size (partial eta squared or η^2^ of 0.06), based on power of 0.80, alpha of 0.05, and a correlation coefficient of 0.5 between repeated measures. Twelve athletes (7 men, 5 women; aged 21.4–28.5 years) participated in the study. Men (age: 24.6 ± 2.2 years, body height: 1.84 ± 0.05 m, body mass: 81.9 ± 5.2 kg, body fat content: 17.8 ± 3.4%, lean leg mass: 10.9 ± 1.1 kg) and women (age: 23.0 ± 1.8 years, body height: 1.65 ± 0.04 m, body mass: 59.4 ± 4.0 kg, body fat content: 24.4 ± 4.8%, lean leg mass: 7.5 ± 0.7 kg) were healthy, with no lower limb injuries in the past six months. All participants trained at least three times per week and had a minimum of five years of athletic training experience across various sports: basketball (n = 2), soccer (n = 1), volleyball (n = 1), jiu-jitsu (n = 2), athletics (n = 3), and contemporary dance (n = 3). The study was approved by the review board at the School of PE and Sport Science of the National & Kapodistrian University of Athens, Athens, Greece (approval number: 1472; approval date: 11 January 2023) and all procedures were in accordance with the Code of Ethics of the World Medical Association (Declaration of Helsinki of 1964, as revised in 2024).

### Design and Procedures

Following thorough familiarization with the test instruments and maximal effort, participants visited the lab on two randomized occasions. On day 1, participants performed dynamic and isometric maximum effort assessments at six knee joint angles (10°, 30°, 50°, 70°, 90°, 110°) using isokinetic dynamometry (ISD) in both seated (90° hip angle) and prone (0° or a neutral hip angle) positions. On day 2, maximum isometric contractions were performed using a hand-held dynamometer attached to a custom-made construction stabilized by two assistants, in the prone position only. Although dynamic testing cannot be replicated using HHD, isokinetic measurements were included to provide a comparative reference for how H/Q ratios may vary depending on the type of muscle action (concentric vs. isometric) across the full range of motion. ISD measurements lasted a maximum of 90 min, while HHD measurements were completed within 30 min. A minimum of 72 h separated the two sessions to ensure recovery from the previous session. The standardized warm-up routine, which preceded all sessions, included 5 min on a stationary ergometer followed by 5 min of lower limb dynamic stretching. Prior to the main tests, participants completed at least three trials for familiarization with both HHD and ISD within 10 days. Measurements were taken for both legs in all assessments. The order of ISD and HHD was randomized and counterbalanced.

### Body Composition Analysis

During the first visit, body composition data were obtained with Dual-Energy X-Ray Absorptiometry (DXA) using the Lunar Prodigy Pro model (General Electric Systems, Madison, WI). Data were analyzed using GE Lunar encore software, version 13.6.

### Evaluation with Isokinetic Dynamometry (ISD)

Participants performed two sets of three maximum repetitions in seated and prone positions using an isokinetic dynamometer (Biodex Medical Systems Inc., New York, USA). Dynamic assessments were conducted at 60°/s, covering a knee range of 0–110° (0° representing full extension). Isometric tests followed 5 min later, with two maximal efforts at six knee angles (10°, 30°, 50°, 70°, 90°, 110°) for both knee flexion and extension (alternating) in randomized and counterbalanced order. The best repetition was recorded for further analysis. A 30-s rest interval was provided between every effort. Dynamometer calibration followed manufacturer guidelines, with gravitational correction applied before each test. Seated tests were performed at a 90° hip angle, with the chest, waist, and thigh straps used to minimize movement. Prone tests included the waist and hip straps, with a pillow under the lower abdomen for comfort. Verbal encouragement was provided during all maximum effort assessments, and the starting position (seated or prone) as well as the starting leg were selected randomly. Peak torque values for concentric flexion and extension were recorded, along with peak torque at the six isometric efforts.

### Evaluation with Hand-Held Dynamometry (HHD)

HHD tests were conducted using a BIOPAC BSL SS25L traction dynamometer (Biopac Systems Inc., Santa Barbara, CA) with a custom-made set-up. Participants were positioned and stabilized on an examination bed in the prone position, similar to the setup of the prone ISD assessment, with straps on the waist and hips. Two assistants held a rigid metal bar, from which the dynamometer was suspended by a small hook. The opposite end of the dynamometer was attached to a strap placed 5 cm above each participant’s outer malleolus, resembling the exact same point of force application in the ISD measurements. For both flexion and extension, assistants held the bar in the opposite direction of the movement to prevent any excess force beyond their strength. At each angle, the bar was adjusted accordingly to ensure that the dynamometer was positioned perpendicularly to the shank. The two assistants adjusted the bar position at each knee angle to ensure measurement accuracy. Knee angles were measured with a BIOPAC SS21L BSL Twin Axis goniometer attached to the knee joint, secured with tape, and calibrated accordingly. Participants performed two maximal isometric contractions at each of the six angles (10°, 30°, 50°, 70°, 90°, 110°) in both flexion and extension, following the same randomization and rest intervals as in the ISD measurements. The efforts were alternated between flexion and extension, and a 30-s rest interval was given between efforts. Once testing on the first leg was completed, the setup was immediately adjusted to proceed with testing on the opposite leg. Verbal encouragement was provided, and the starting leg was randomized.

### Data Acquisition and Analysis

ISD data were acquired at 100 Hz and then exported to excel files. In HHD testing, the dynamometer was connected to a BIOPAC MP35 data collector (1000 Hz), synchronized with the goniometer, and analyzed using Acknowledge 4.2.0 software (Biopac Systems Inc., Santa Barbara, CA). Since the HHD setup lacked precise angle control, polynomial fitting (3rd-degree) was applied to the six goniometer-derived angles, allowing exact force determination at the six knee angles (10°, 30°, 50°, 70°, 90°, 110°) for comparison with isometric ISD measurements. To reduce measurement error, the average of two HHD efforts was used instead of the maximum. The HHD data were compared with the ISD data for both concentric and isometric measurements in seated and prone positions.

Conventional H/Q ratios were calculated by dividing peak flexion torque by peak extension torque, irrespective of the knee angle at which they were attained. Angle-specific H/Q ratios were calculated by dividing the flexion by the extension torque values at each individual knee angle (10°, 30°, 50°, 70°, 90°, 110°).

### Statistical Analysis

Two-way ANOVAs for repeated measures compared dominant and non-dominant leg results across knee angles (2 legs x 6 angles) for flexion and extension separately. Two-way ANOVAs for repeated measures were also used to compare peak torque values at each knee angle and angle-specific H/Q ratios across the five conditions (5 conditions x 6 angles) for flexion and extension separately. One way ANOVA for repeated measures was used to compare the conventional H/Q ratios across the five conditions. Post-hoc analyses were performed using the Tukey’s HSD test. Results were expressed as mean ± standard deviation, with effect sizes (η^2^) and reliability (ICC) reported. Significance was set at *p* < 0.05. All statistical analyses were performed using SPSS software (version 26.0, IBM Software).

## Results

No significant differences were observed

between dominant and non-dominant legs in torque for all knee angles (average *p* for all comparisons: 0.59 and 0.48, for main effect and interaction, respectively). As a result, data from the dominant leg were used for further analysis.

### Reliability and Validity Analysis

**Figure 1:**
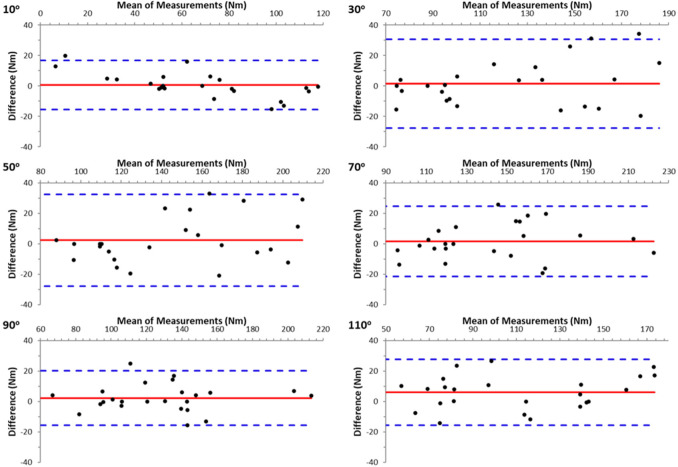
Bland-Altman plots showing the agreement for knee extension torque values between two hand-held dynamometry measurements, for the six knee angles.

**Figure 2:**
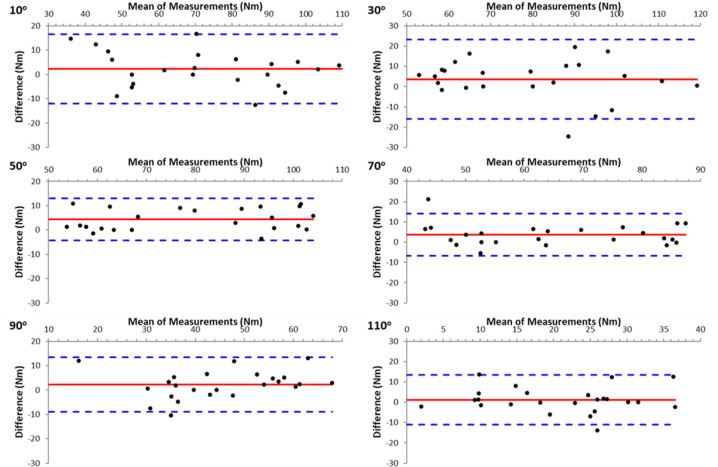
Bland-Altman plots showing the agreement for knee flexion torque values between two hand-held dynamometry measurements, for the six knee angles.

The results demonstrated high reliability between the two trials in all measurements. In ISD measurements, reliability was particularly high between the two attempts at all angles of the isometric evaluation, both in the seated and prone positions, for knee flexion and extension (ICC = 0.955–0.993, *p* < 0.001). HHD data also demonstrated high reliability at all angles for knee flexion and extension (ICC = 0.812–0.971, *p* < 0.001). The agreement between the two repetitions at each knee angle for knee extension and knee flexion (Figure 2) is displayed in the form of Bland-Altman plots (Figures 1 and 2, respectively). As can be seen the agreement was good and there was no bias in HHD measurements.

### Knee Extension and Flexion Torques

**Figure 3:**
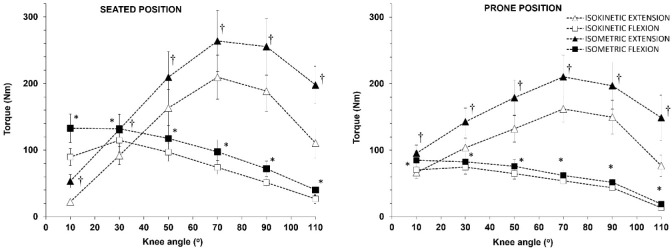
Peak extension and flexion torque vs. knee angle relationships measured on the isokinetic dynamometer in the seated (left panel) and the prone position (right panel) concentrically (Isokinetic at 60 ^o^/s) or isometrically. * p < 0.001 from the isokinetic flexion values; †: p < 0.001 from the isokinetic extension values

Figure 3 shows torque values per knee angle in the seated and prone positions for knee extension and flexion. As expected, the isometric torque-knee angle curves were higher than the concentric ISD torque for both flexion and extension (*p* < 0.001, η^2^ = 0.58 to 0.88). Specifically, isometric knee extension torque values were approximately 32–40% higher than corresponding ISD torque values (*p* < 0.001, η^2^ = 0.74 to 0.88), while isometric knee flexion torque values were approximately 17–31% higher than corresponding ISD torque values (*p* < 0.002, η^2^ = 0.58 to 0.72) (Figure 3).

**Figure 4:**
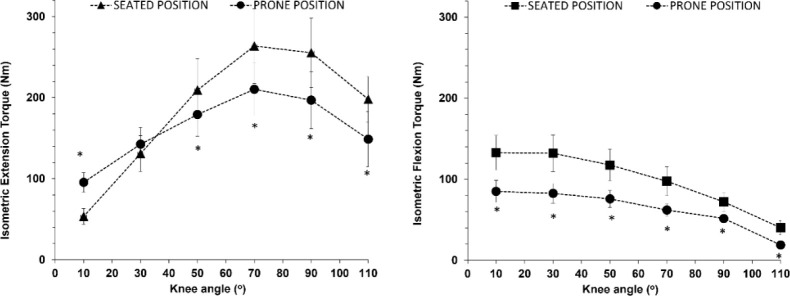
Peak extension (left panel) and flexion (right panel) torque vs. knee angle relationships measured isometrically on the isokinetic dynamometer in the seated and the prone position. * p < 0.001 from the corresponding values in the seated position

There was an interaction between the position and the knee angle for both isometric and isokinetic measurements for knee extension (seated vs. prone, *p* < 0.001, η^2^ = 0.66 to 0.52), indicating that the torque vs. knee angle relationship shifted to the left and downwards when measured in the prone position compared to the seated position (Figure 3). Post-hoc comparisons showed that for knee extension, peak torque in the prone position was higher than the corresponding torque measured in the seated position when measured close to knee extension (knee angle of 10^o^), similar at the knee angle of 30^o^ and lower at the knee angles of 50–110^o^ (Figure 4, left panel). For knee flexion, peak torque in the prone position was by approximately 35% lower than the corresponding torque measured in the seated position at all angles (Figure 4, right panel).

**Figure 5:**
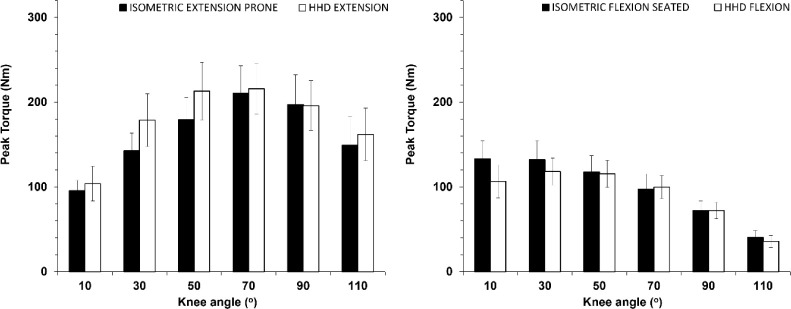
Comparison between peak extension (left panel) and peak flexion (right panel) torque vs. knee angle relationships measured isometrically on the isokinetic dynamometer in the seated and the prone position, and isometrically using the hand-held dynamometer (HHD).

The comparison between HHD and ISD isometric measurements in the prone position showed that for knee extension there was no significant main effect for the position or interaction (*p* > 0.38, Figure 5, left panel). Furthermore, there was no main effect for the position between HHD and the seated ISD isometric measurements (*p* = 0.48), although there was a position vs. angle interaction (*p* < 0.01). The post-hoc tests showed that the only difference between HHD and isometric peak torque in the seated position was at the knee angle of 10^o^.

For knee flexion, there was no main effect of the position or a position vs. angle interaction between HHD and the seated ISD isometric measurements (*p* > 0.48, Figure 5, right panel). In the prone position, there was both a main effect of the position (*p* = 0.86) and an interaction (*p* = 0.23). The post-hoc tests showed that peak torque was higher when assessed with HHD compared with ISD isometric measurements in the prone position (*p* < 0.01), except the knee angle of 110^o^ where values were equal.

### H/Q ratios

Conventional H/Q ratios were similar in HHD and isometric and concentric ISD measurements in the seated position (56 ± 11%, 51 ± 10% and 55 ± 8%, respectively, *p* > 0.792). However, H/Q ratios in the prone position during ISD measurements were lower than the corresponding seated values when measured either in the isometric (41 ± 8%, *p* < 0.03) or in the concentric modes of ISD (46 ± 8%, *p* = 0.03).

Angle-specific H/Q ratios were similar across all modes of measurements and knee angles (*p* > 0.70), with the exception of the knee angle of 10^o^, which was the position where the knee was almost extended (Table 1). In the isokinetic and isometric modes of measurements in ISD, angle-specific H/Q ratios in the prone position differed from the seated position only at the knee angle of 10^o^ (Table 1). The highest H/Q ratios were observed at the knee angles of 10^o^ and 30^o^, with values leveling off after 50^o^ of knee flexion (Table 1).

**Table 1:** Knee angle-specific H/Q ratios in the different modes of measurement. Values are means ± SD.

		Knee angle (degrees)						
MODE	10		30	50	70	90	110
CON SEATED	459% ± 195%**		127% ± 28%	62% ± 14%	37% ± 8%	29% ± 8%	30% ± 24%
CON PRONE	118% ± 66%††		74% ± 19%	50% ± 11%	34% ± 8%	32% ± 9%	19% ± 12%
ISO SEATED	268% ± 90%**		104% ± 23%	59% ± 13%	38% ± 9%	30% ± 9%	21% ± 9%
ISO PRONE	90% ± 22%††		59% ± 13%	43% ± 6%	31% ± 8%	30% ± 12%	18% ± 16%
HHD	112% ± 39%		72% ± 26%	57% ± 16%	47% ± 8%	38% ± 9%	24% ± 10%

CON: concentric, ISO: isometric, HHD: hand-held dynamometry, **: p < 0.001 between HHD and the corresponding mode of measurement and knee angle. ††: p < 0.001 from the ISO seated and CON seated positions at the corresponding knee angle

## Discussion

The aim of this study was to examine the feasibility, reliability and accuracy of a simple, custom-built system for measuring knee flexor and extensor strength across the full ROM. Additionally, this study aimed to provide novel data on angle-specific H/Q ratios at different hip joint positions and types of muscle contraction. The main findings include the high accuracy and reliability of HHD in measuring knee extension and flexion strength in the prone position compared to the gold standard ISD, the similarity of angle-specific H/Q ratios between HHD and all modes of testing in all knee angles, except the angle of 10^o^, and the significant influence of the hip flexion angle on torque and conventional H/Q ratios.

One key finding of the study was that HHD may be used as an accurate and low-cost alternative for evaluating knee extension and flexion strength and H/Q ratios on an examination bed using minimal and low-cost equipment. The results confirmed that HHD isometric knee extension measurements closely matched ISD measurements in the prone position. This supports prior research validating HHD against ISD at a single joint angle, when the participant’s position is closely controlled (Bohannon, 1990). Previous studies using different body positions (e.g., supine and seated) have similarly shown moderate to strong correlations between HHD and ISD at a single angle (Li et al., 2006; Martin et al., 2006; Piao et al., 2004), though limitations regarding assessor strength remain (Deones et al., 1994), especially when testing athletic populations (Whiteley et al., 2012). In the present study, these limitations were mitigated by an efficient setup that included two assistants stabilizing the system. ISD concentric knee extension values were notably lower than both HHD and ISD isometric measurement results (Figure 3) due to expected reductions in force production with a higher angular velocity, in line with the force-velocity relationship (Thorstensson et al., 1976).

An interesting observation in the present study was that peak extension torque in the prone position was lower than that in the seated position at larger knee angles, with the knee angle vs. torque relationship indicating a downward and leftward shift (Figure 4). This has been observed in the past (Deighan et al., 2012; Pavol and Grabiner, 2000) and may be explained by changes in the force-length relationship of the biartucular rectus femoris muscle, and differences in muscle activation when the hip is in the neutral (close to 0^o^) position (Hasler et al., 1994; Salzman et al., 1993). HHD torque data were identical to ISD isometric data in the prone position, validating our approach (Figure 5, left panel). However, HHD isometric knee flexion values aligned more closely with ISD isometric values in the seated position (Figure 5, right panel). This may be due to subtle postural adjustments during HHD, such as participants raising their hips during their effort, which may alter the hip angle to resemble a seated position. This minimal movement led to an increase in the hip angle which potentially explains the HHD results being similar to the ISD seated isometric efforts. This effect is consistent with recent findings indicating that knee flexion torque decreases when the hip angle is close to neutral (10°) compared to a flexed hip position (90°) (Baumgart et al., 2021). Previous research also supports that flexor torque decreases as hip extension increases (Deighan et al., 2012; Guex et al., 2012). This is because when the hip is extended, the length of the hamstrings decreases, as their origin moves closer to their insertion. In contrast, the length of the biarticular rectus femoris increases as the anterior inferior iliac spine moves away from the tibial tuberosity, thus slightly elongating the muscle-tendon unit. This modifies the resistance to flexion in the prone position, across the whole ROM and especially towards knee flexion, as previous studies have also demonstrated (Ema et al., 2017; Maffiuletti and Leppers, 2003).

Conventional H/Q ratios were lower in the prone position, potentially due to the reduction in muscle length of the hamstrings (Kellis and Blazevich, 2022) and the increase in muscle length of the rectus femoris (Ema et al., 2017). Conventional H/Q ratios were consistent between HHD (56%) and ISD in concentric and isometric seated measurements (50–55%), while ratios in prone ISD were significantly lower (41–46%) due to the anatomical and mechanical differences mentioned previously. The angle-specific H/Q ratios were also found to escalate with a decreasing knee angle in all modes of measurement, and this effect was amplified in the seated position and concentric measurements. These findings challenge the common practice of using standard 55–66% thresholds for knee flexor-extensor imbalance detection (Heiser et al., 1984) and suggest the need for comprehensive assessments that consider the joint angle and the contraction type. The results align with Baumgart et al. (2021) who demonstrated that hip flexion angles influenced torque generation inconsistently across ROM, with H/Q ratios starting near 100% in early ROM and declining to 30–40% at full ROM. A recent review on the force-length relationship at different hip and knee angles reported that knee flexor torque increased with hip flexion, but knee extensor torque remained almost stable across hip angles (Kellis and Blazevich, 2022). For instance, Guex et al. (2012) found that knee extensor peak torque did not vary significantly across all hip flexion angles, whereas knee flexor peak torque was lowest at 0° of hip flexion relative to any other angle and highest at 90° of hip flexion, especially in dynamic assessments. Furthermore, they observed that as the hip angle increased, the H/Q ratio also increased. This stresses that tests conducted in seated positions cover only a limited ROM, excluding a functional range where hamstring torque potential is maximized. Conversely, prone or supine knee flexion positions restrict the optimal torque development range for hamstrings due to limited hip flexion angles (Kellis and Blazevich, 2022). In any case, the determination of conventional or angle-specific H/Q ratios using HHD was proven to be a valid and reliable method, providing similar values across almost all knee angles in all modes of measurement.

One limitation of this study is the challenge of measuring knee flexion using HHD in the prone position. Despite appropriate strapping, hips were not stabilized completely, thus resulting in a slight change of the hip angle which affected the torque measured. Indeed, the torque curve in the prone position was closer to that measured in the seated than the prone ISD position. Additionally, the unique setup with the participants lying in the prone position may limit the applicability of the findings to other populations, such as overweight and injured individuals. However, measurements with the hip in the more extended position (close to 10^o^ of flexion) have been characterized as more ecologically valid, compared to the seated measurements (Deighan et al., 2012). Another limitation was the use of two assistants to stabilize the system; however, this approach may be suitable in sports settings, since stabilization does not require expertise and may be attained by other personnel. Importantly, this measurement system is inexpensive and portable, allowing measurements to be performed at any location, since the goal was to create an affordable, accessible solution for strength testing in environments with limited resources. Future research could improve hip stabilization and include a larger sample size to enhance the reliability of the findings.

## Conclusions

This study confirms that hand-held dynamometry (HHD) is a reliable, cost-effective and efficient method for measuring knee flexor and extensor strength, demonstrating that HHD values for knee extension in the prone position are comparable to those obtained via isokinetic dynamometry (ISD) in the same position, while knee flexion results align more closely with ISD measurements in the seated position. Notable differences in strength values across various positions can be attributed to anatomical factors, such as hip positioning affecting muscle length and torque generation. Additionally, the similarity of conventional H/Q ratios between HHD and seated isometric and isokinetic measurements adds to the value of this approach. Finally, the investigation of angle-specific H/Q ratios reveals that conventional benchmarks do not accurately represent muscle imbalances, underscoring the necessity for tailored assessments based on joint angles. This study provides critical insights into the importance of considering hip flexion angles during strength evaluations, with practical applications in both sport and potentially clinical contexts.
